# Airborne particulate matter from biomass burning in Thailand: Recent issues, challenges, and options

**DOI:** 10.1016/j.heliyon.2023.e14261

**Published:** 2023-03-05

**Authors:** Phuchiwan Suriyawong, Santi Chuetor, Hisam Samae, Suthida Piriyakarnsakul, Muhammad Amin, Masami Furuuchi, Mitsuhiko Hata, Muanfun Inerb, Worradorn Phairuang

**Affiliations:** a Research Unit for Energy, Economic, And Ecological Management (3E), Science and Technology Research Institute, Chiang Mai University, Chiang Mai, 50200 Thailand; b Department of Chemical Engineering, Faculty of Engineering, King Mongkut's University of Technology North Bangkok, Bangkok, 10800 Thailand; c Office of National Higher Education Science Research and Innovation Policy Council, Bangkok 10330 Thailand; d Faculty of Geosciences and Civil Engineering, Institute of Science and Engineering, Kanazawa University, Kakuma-machi, Kanazawa, Ishikawa, 920-1192 Japan; e Faculty of Engineering, Maritim University of Raja Ali Haji, Tanjung Pinang, Kepulauan Riau 29115, Indonesia; f Faculty of Environmental Management, Prince of Songkla University, Hat Yai, Songkhla, 90110, Thailand; g Department of Geography, Faculty of Social Sciences, Chiang Mai University, Muang, Chiang Mai 50200 Thailand

**Keywords:** Air quality, Biomass combustion, Emission source, Human health, PM_2.5_

## Abstract

Many of the current atmospheric environmental problems facing Thailand are linked to air pollution that is largely derived from biomass burning. Different parts of Thailand have distinctive sources of biomass emissions that affect air quality. The main contributors to atmospheric particulate matter (PM), especially the PM_2.5_ fraction in Thailand, were highlighted in a recent study of PM derived from biomass burning. This review is divided into six sections. Section one is an introduction to biomass burning in Thailand. Section two covers issues related to biomass burning for each of the four main regions in Thailand, including Northern, Northeastern, Central, and Southern Thailand. In northern Thailand, forest fires and the burning of crop residues have contributed to air quality in the past decade. The northeast region is mainly affected by the burning of agricultural residues. However, the main contributor to PM in the Bangkok Metropolitan Region is motor vehicles and crop burning. In Southern Thailand, the impact of agoindustries, biomass combustion, and possible agricultural residue burning are the primary sources, and cross-border pollution is also important. The third section concerns the effect of biomass burning on human health. Finally, perspectives, new challenges, and policy recommendations are made concerning improving air quality in Thailand, e.g., forest fuel management and biomass utilization. The overall conclusions point to issues that will have a long-term impact on achieving a blue sky over Thailand through the development of coherent policies and the management of air pollution and sharing this knowledge with a broader audience.

## 1 Introduction

Air pollutant emissions from biomass burning in Thailand usually focus on particulate matter (PM) emissions and their chemical compositions in the past decade [[1], [2], [3], [4]]. The PM concentrations observed in Thailand are based on a range of coarse particles (PM_10_) to fine particles (PM_2.5_) [[5], [6], [7], [8], [9]] and, to some extent, to sub-micron particles (PM_1.0_) and ultrafine particles (PM_0.1_) [2,10,11]. This review is comprised of a collection of recently published papers related to all aspects of biomass burning in Thailand. Over 135 peer-reviewed journals that mostly appeared in Scopus and Web of Science databases over the past five years (2018–2022) were used to analyze and integrate this subject. The keywords that were searched included “biomass burning, Thailand, particulate matter, biomass utilization, haze pollution, and health effects. The six sections of this contribution include the following: section one is an introduction to biomass burning in Thailand. The second section covers issues concerning biomass burning for each of the four main regions in Thailand, i.e., Northern, Northeastern, Central, and Southern Thailand. The third section reviews the effect of biomass burning on human health. Finally, perspectives, new challenges, and policy recommendations are made concerning improving air quality in Thailand, e.g., forest fuel management and biomass utilization.

## 2 Background and significant impact of biomass burning

Biomass burning (BB) involves the burning of dead and living creatures, including crop residues and forest-derived materials [12,13]. The air pollutant from burning vegetation contains high levels of ambient particulate matter (PM) [[14], [15], [16]]. It is largely comprised of toxic gaseous and particulate-phase, i.e., sulfur dioxide (SO_2_), carbon monoxides (CO), nitrogen oxides (NO_x_), and polycyclic aromatic hydrocarbons (PAHs) [5,7,[17], [18], [19]]. The number of pollutants emitted from BB causes haze problems in many countries [[20], [21], [22], [23]]. The major pollutants that are produced from biomass combustion are PMs; approximately 80–90% of the mass concentration is PMs down to the accumulation mode (diameter <1 μm) [[24], [25], [26]]. Lesser mass fractions of around 10% contain coarse particles (diameter of 2.5–10 μm), and a minor fraction of the coarse fraction contains ash particles (2 < diameter <20 μm) [27]. It is important to note that open BB is a significant contributor to the production of ultrafine particulate matter (PM_0.1_, or nanoparticles) [3,28,29]. Conversely, the PM size distribution depends on the fuel type, moisture content, and the combustion method, among others [24,30,31]. The combustion process can be divided into smoldering and flaming phases, which are dependent on combustion efficiency, which is the part of fuel burned by ambient O_2_ levels and CO/CO_2_ ratios [25,26]. Additionally, emissions from biomass burning contain a significant carbon content, including organic carbon (OC) ∼ 40–50% and black carbon (BC) ∼5–10% [32,33]. Smoke particles from biomass combustion affect the global atmosphere through the absorption and reflectance of solar radiation [[34], [35], [36]].

PMs emitted from biomass combustion affect the atmosphere by having both a direct and an indirect effect on the extent of atmospheric radiation [22,[36], [37], [38]]. The immediate impact of PM is absorbing and scattering solar radiation, which influences global climate change [39,40]. Indirect effects include the accumulation of cloud condensation nuclei (CNN) that increase the cloud albedo [41]. The PM from biomass burning can have a severe effect on human health, including cardiovascular morbidity, respiratory symptoms, and adult mortality in high-risk groups [[42], [43], [44], [45]]. Smaller particles from BB, especially fine and ultrafine particles, can travel deep into the lung - mainly in the alveolar region [46]. Correspondingly, hazardous PM chemicals, such as carcinogenic PAHs and heavy metals, directly affect human health [3,47,48].

Thailand is an agricultural-based country that produces a large amount of biomass residues that are frequently burned in the field or by agro-industries (outfield) to generate energy and electricity [12,49]. BB in the area is also done to prepare for the next crop cycle and remove weeds, insects, and animals. Open burning is a conventional method and an easy way to eliminate residues on small farms in agricultural zones [50,51]. Managing crops by open burning is widespread in Thailand and other developing Asian countries, i.e., China, India, and Myanmar [18,49]. In addition, the agro-industry continues to be a traditional process in many countries, including Thailand. Industrial production processes are associated with a large amount of energy consumption and the generation of organic waste, and burning is a standard method for recycling waste materials that are produced [52,53]. After processing, the biomass is turned into an energy source as feedstock for boilers [54,55]. In the agro-industry sector, biomass is one of the primary energy sources for agro-processing [56]. However, energy consumption and the related emissions of pollutants in the area are considerable, i.e., crop residue burning and biomass fuel utilization is a direct cause of large amounts of PM emissions, which are not easily controlled [19,24].

**Fig. 1:**
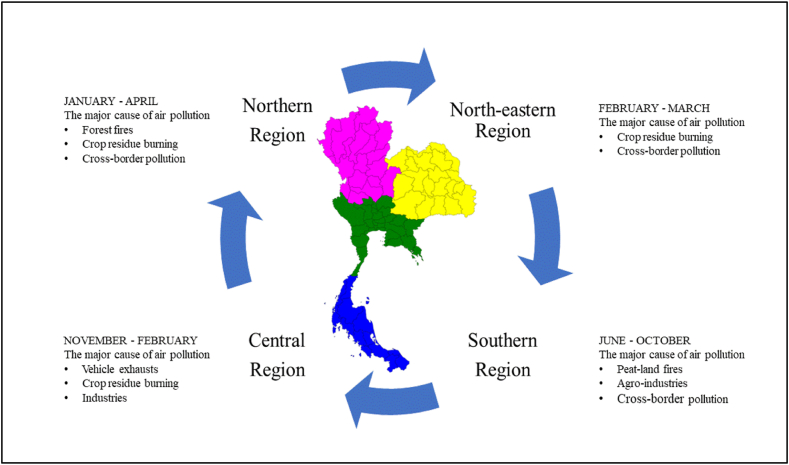
Overall air pollution in Thailand.

Fig. 1 displays the overall air pollution in Thailand. In northern Thailand, open biomass burning, including agricultural residue burning and forest fires during the dry season from January to April, appears to have played a vital role in air quality in the past decade [1,2,57]. Moreover, in the northeast part of Thailand, pre- and post-harvesting of crops, e.g., rice and sugarcane, is the main contributor to PM in this region during the dry season [58]. In Southern Thailand, the impact of maritime aerosols, biomass combustion, and possible crop residue burning is the primary source of PM_10_ from June to October nearly every year [6]. However, in the Bangkok Metropolitan Region (BMR) in the central part of Thailand, the main contributors to air pollutants are from motor vehicles, industries, open biomass burning, and secondary pollutants [2,18]. Therefore, the influence of forest fires, crop residue burning, and agro-industries are the main contributors to ambient PMs (coarse and fine mode particles) in Thailand [12]. Nevertheless, emissions from land transportation and industries in an urban area, i.e., BMR, are an essential source of particles [50,59].

In Thailand, the emission inventory (EI) of PM_10_ included biomass burning (40%), industrial activities (32%), traffic emission (17%), and power plants (11%). Different parts of Thailand have different sources of air pollutants [60]. Cross-border particulate pollution is one of the important issues in atmospheric pollution studies in southeast Asia [[61], [62], [63]]. However, there is a lack of studies concerning the spatiotemporal characteristics of aerosols that are produced in Thailand, particularly the PM_1.0_ and PM_0.1_ fractions. Hence, the physical and chemical characteristics of PMs in Thailand need to be investigated with emphasis on the primary emission sources and air quality management in the near future [2].

## 3 Biomass burning in different parts of Thailand

### 3.1 Biomass burning in the northern part of Thailand

**Fig. 2:**
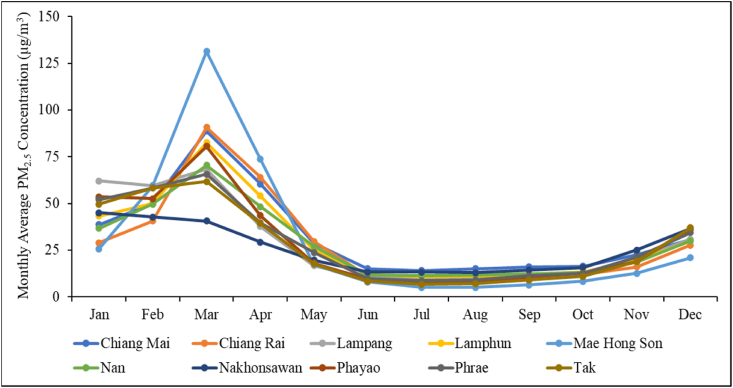
The monthly average PM_2.5_ concentrations in the northern province of Thailand.

Air pollution has been a serious environmental problem worldwide because of its potential impact on human health [[43], [44], [45],^64^]. Northern Thailand is confronted with the problem of air pollution in nearly every dry season from January to mid-April [2,65]. Fig. 2 illustrates the monthly PM_2.5_ concentrations obtained from the Pollution Control Department (PCD) in northern Thailand at each province station based on a 3-year average (2019–2021). The PM_2.5_ concentrations are clearly increased during every dry season (January–April). Typically, they start to rise around mid-January and reach a peak in March where they then decrease. These areas of burning include open fires, including forest fires and crop waste burning [12,67,68] and terminate by mid-April [66].

The above information corresponds to the number and locations of fire hotspots, which are high in the dry season and low in the rainy season [69,70]. Each hotspot/active fire location represents one or more actively burning hotspots/fires within that pixel. The latest decade's famous satellite data is the Moderate Resolution Imaging Spectroradiometer (MODIS), a satellite-based sensor used for earth and climate measurements [10,71,72]. MODIS has two main satellites, the Terra (originally known as EOS AM-1) and Aqua (originally known as EOS PM-1). Terra's orbit around the Earth is timed so that it passes from north to south across the equator in the morning, whereas Aqua passes south to north over the equator in the afternoon. These orbits can be used to detect fire hot spots in Thailand [73]. Moreover, these numbers of hot spots are closely correlated with the concentration of particulate matter [10,12]. Additionally, stable meteorological conditions, such as low wind speed and temperature inversions, favor the accumulation of pollutants in the lower atmosphere, thus limiting the pollutants' dispersion. In addition, the geography is a basin area surrounded by high mountains supporting an increased occurrence of air pollutants in the area [9,[74], [75], [76]].

### 3.2 Biomass burning in the northeastern part of Thailand

Biomass burning derived-PM in the northeastern part of Thailand is associated with agricultural activities, and the pre-and post-harvesting of crops is this region's primary source of ambient PM. As reported by Kumar et al. (2020) [58], around 83% of the total burnt crop residue in Thailand arises from rice and sugarcane, especially in northeastern Thailand, which represents a large area for cultivation. In developing countries, especially in Southeast Asia, open biomass burning is a common way to clear the crop before or after harvesting. Moreover, this removes agricultural residues and controls weeds after harvesting [49].

**Fig. 3:**
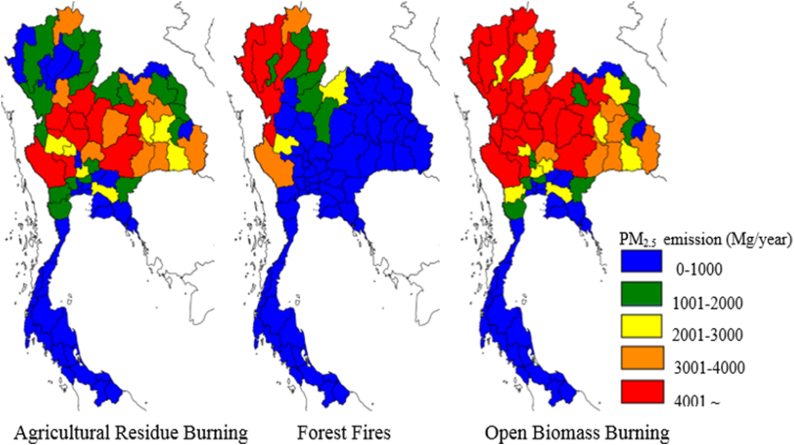
PM_2.5_ from Biomass burning in Thailand adapted from [12].

In the northeast of Thailand, the important economic agro-industry crop is sugarcane, which is increasing rapidly because the Thai government promotes renewable energy, e.g., bioethanol and gasohol. The total production has been high in Thailand and has rapidly expanded from 2010 to 2019. The total sugarcane output in Thailand has increased from 49.58 million tons in 2010 to 128.53 million tons in 2019 [77]. Sugarcane production has increased rapidly by more than 100% in the past ten years. Most of this effort has been directed to the production of two main products, namely, sugar and molasses [78]. An enormous amount of biomass fuel is produced during the processing of sugarcane and these residues are typically used as an energy source. This material is readily available, low cost, and can be used directly. In contrast, energy consumption related to pollutant emissions in the sugarcane production process may contribute to the total air pollutants, such as PMs, since they are not controlled in many cases [53]. The total PM_2.5_ emission from biomass burning in Thailand was released mainly from the Northeast and North part, followed by the Central and South region, respectively (Fig. 3). The extensive agricultural residue burning in the northeast generates considerable PM and greenhouse gases [79]. Furthermore, sugarcane is a crucial economic crop in lower northern, central (excepting BMR), and Northeastern Thailand. The northeast is the main area that emits PM from crop biomass fires into the atmosphere [12]. However, only limited ground-based information is currently available for the northeast of Thailand.

Information concerning air pollution in the northeast region is still limited compared to other parts of Thailand. Salao et al. (2021) [80] reported on the PM_2.5_ fraction in the ambient air of Muang District, Ubon Ratchathani Province. They found that biomass burning and exhausts from motor vehicles were significant contributors to the carbonaceous component in PM_2.5_. Moreover, Sakunkoo et al. (2022) [81] reported that the PM_2.5_ fraction is related to the risk to human health in Khon Kaen Province. They found that Biomass combustion is a primary source of PM_2.5_ mass concentrations at residential sites due to local and transboundary particulate pollutants in this area.

### 3.3 Biomass burning in the central part of Thailand

The central part of Thailand includes the Bangkok Metropolitan Region (BMR). Almost every year in Thailand, during the dry season (November–February), the high-pressure conditions result in higher dust concentrations than normal. There is also the phenomenon of inverse temperature and calm weather and wind speed that causes smoke and dust to accumulate in this area, which is a serious problem [82]. The sources of fine particles emitted in BMR were identified as being from traffic, biomass burning, sea spay, power plants, and industries at 44%, 24%, 11%, 7%, and 4%, respectively [5]. Moreover, recent studies by Phairuang et al. (2022) [83] reported that smaller particles, or nanoparticles (PM_0.1_), mainly arise from land transportation. Except during the dry season, from November to February in the following year, biomass burning in both local and transboundary cross-country areas increases the PM_0.1_ particle concentration in BMR.

The central part of Thailand is a large area for rice and sugarcane cultivation and extends along with the Chaopraya basin [18]. PMs from agricultural activities can be transported to BMR within 24 h, depending on the wind speed and direction [12]. The apportionment of the source of these particles in a case study of BMR indicated that the source of PM_2.5_ in BMR during the period 2015 to 2017 was biomass burning up to 35% in Bangkok and 38% at the Asian Institute of Technology (AIT) during the dry season. Compared to the wet season, the values were 25% for Bangkok and 25% for AIT [84]. Choomanee et al. (2020) [85] studied the vertical profile of fine particles related to carbon composition in the atmosphere of Bangkok. They found that particle concentration in ambient air changes with elevation. The concentration of fine particles increased with increasing elevation and, in contrast, carbon concentrations decreased with increasing elevation. Bangkok's ambient air is mainly influenced by pollution from land transport and car engines are considered to be the primary source of PM_2.5_. However, at 110 m above ground level, the likely source would be biomass combustion from a neighboring area [85,86].

### 3.4 Biomass burning in the southern part of Thailand

**Fig. 4:**
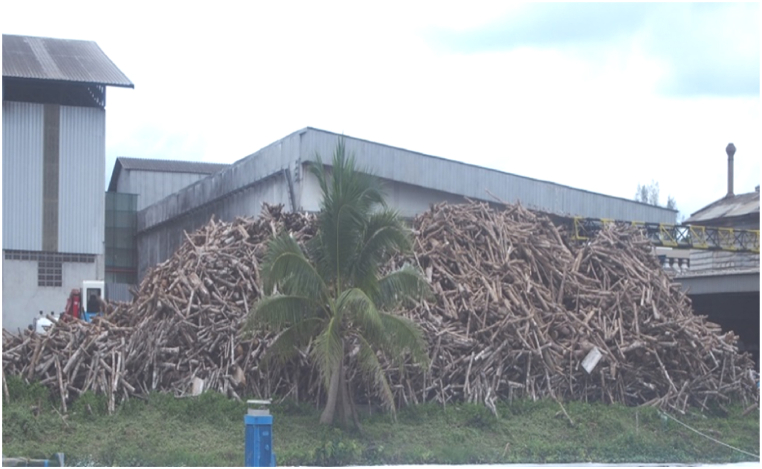
Para-rubber fuelwood used in agro-industries in southern Thailand.

Southern Thailand is different from the other parts of Thailand. Climate weather conditions and economic crops are generally planted in this region, and para-rubber and oil palm trees are the main plantations in southern Thailand [87]. Many of the plant residues in this area are used as the primary fuel for agro-industries, such as rubber and oil palm factories [24]. It is estimated that there are 1869 factories in Songkhla province, the capital economic city in southern Thailand. A total of 1255 factories (67%) are agro-industry based operations [19]. These factories mainly use fuel derived from biomass residues from para-rubber production. The main agroindustry using fuelwood produces ribbed smoke sheet rubber (RSS). These results indicate the environmental load due to hazardous air pollutants such as smoke particles, and carcinogenic PAHs is now a serious problem in southern Thailand [19,24]. Hat Yai, the economic capital of Songkhla province, is a main city in the south of Thailand. It has been reported that the primary airborne pollutants in Hat Yai are caused by biomass combustion from the various rubber industries [88]. The moisture content of wood and the time of burning have an effect on smoke particles and associated PAHs that are produced in the workplace and surrounding areas [24]. Fig. 4 shows the use of para-rubber fuel by the agro-industries in southern Thailand.

Pongpiachan et al. (2014) [89] reported that marine aerosols, biomass combustion, and possible crop residue burning are the primary source of PM_10_-bound carbon components on the top of a building in Hat Yai city, an economic center city in the southern part of Thailand. Phairuang et al. (2017) [12] reported that open BB is not a vital source of ambient PMs in southern Thailand. However, BB by the industrial sector should be considered to be the primary contributor of aerosols in the region. Interestingly, recent research by Choochuay et al. (2020) [6] and Phairuang et al. (2020) [23] indicated that transboundary atmospheric PM from Indonesian forest fires is a significant factor in increasing the levels of PMs during the dry season in lower southeast Asia. Atmospheric PM_2.5_ on Phuket Island from March 2017 to February 2018 was 42.3 μg/m^3^ annually. The average PM_2.5_ mass concentrations in Phuket significantly exceeded Thailand's ambient air quality standards for one year-PM_2.5_. The primary emission source was biomass burning, diesel engines, sea spray, and the industrial sector [6].

Similarly, Phairuang et al. (2020) [23] examined the size-fractionated particulate matter down to PM_0.1_ related to carbon compositions over 1 year in 2018. The results suggested that solid biomass burning from agro-industries contributes to the particle-bound carbon in the total PM concentration over Hat Yai city, in southern Thailand. The ambient nanoparticles (PM_0.1_) mainly arose from road transportation [90]. However, during the pre-monsoon season (June–August), the carbon content in the PM_0.1_ fraction may increase from the long-range transport of particles produced by large open BB in Indonesia. However, southern Thailand's local open burning is slight compared to other regions [12]. Moreover, there have been forest fires in peat-land areas since July–August. It is generally thought that these forest fires are human-made, i.e., land clearing before crop cultivation and collecting forest products (i.e., honey) [91].

Overall, the influences of solid fuel wood in agro-industries are the main contributor to the ambient PMs in southern Thailand. Traffic emissions in urban areas are significant in the normal period, and the local open BB is unnecessary. However, the long-range transport of aerosols from Indonesian peat-land fires may affect the air quality in lower southern Thailand during the pre-monsoon season, depending on emission sources, weather conditions, and wind direction [47,92].

## 4 Effects of biomass burning on public health in Thailand

Industrialization and urbanization have increased in Thailand in recent years. PM from biomass burning can increase human health risks in the general population, particularly during periods of haze in the dry season [93]. Fifteen peer-reviewed research articles published between 2018 and 2022 reported on health impact assessments of the BB-derived PM in Thailand, as summarized in Table 1. Most of the published studies dealt with the effect of smoke haze on the respiratory system, mainly in northern Thailand, which is exposed to open biomass burning every year. In the northern part of Thailand, it has long been recognized that biomass burning has an effect on human health, both short-term and long-term. Pothirat et al. (2021) [94] reported that the mortality of people in the northern part is related to the increased particle concentration during the dry season. However, the long-term effects on public human health continue to be challenging. It is well known that PM_2.5_ induces respiratory illnesses such as bronchitis, asthma, and emphysema [95]. Taking into account the smaller particles, e.g., nano-scale size, this has increased the concerns regarding health risks; however, data on PM_0.1_ fraction derived from biomass fires are still limited worldwide. It is interesting that Chiang Mai, an economically important city in northern Thailand, has a high level of ambient ultrafine particles during the dry season that result from substantial forest fires and agricultural residue burning [2,3].

**Table 1:** Overview of studies related to the health impact of biomass burning in Thailand.

Study	Location	Type of location	Duration	Health risk	Major findings
Kongpran et al. (2021) [96]	Northern Thailand	Urban and Rural	March and July 2018	The harmful effects of PAHs are dependent on Benzo[a]pyrene (BaP) via inhalation partway	The gas and particulate phases of PAHs during the haze and non-haze associated with 926 and 25 per million cancer cases, respectively.
Mueller et al. (2020) [97]	Northern Thailand	Urban	2014–2017	Ischaemic heart disease (IHD), Chronic lower respiratory disease (CLRD), and cerebrovascular disease (CBVD)	The peak biomass fires are the highest PM_10_ levels in March, concurring with daily PM_10_ and patient visits, and raised up most on the same day as exposure for CLRD = 1.020 and CBVD = 1.020, with no linked to IHD.
Mueller et al. (2021) [98]	Northern Thailand	Urban	2015–2018	Biomass burning related to birth weight (BW) and low birth weight (LBW) by semi-ecological analysis	The whole pregnancy exposure related to reduced BW both for PM_10_ (−6.81 g per 10 μg/m^3^ increase in PM_10_ and biomass combustion (−6.34 g per 1 SD increase in fires/km^2^
Niampradit et al. (2022) [99]	Chiang Rai (North)	Urban	Haze (March 2021) and non-haze episodes (July–August 2021)	PM_2.5_-bound 10 trace elements (K, Mn, Fe, Cu, Cr, Cd, Ni, Pb, Zn) linked to health risks in both carcinogenic and non-carcinogenic	The non-carcinogenic measures in the tolerable risk assessment range in general populations. However, for carcinogenic risk, Chromium (Cr) surpassed the safety level.
Pani et al. (2020) [22]	Northern Thailand	Urban	March–May 2016	Risk of lung cancer (LC), The health risk estimates of equivalent black carbon cardiovascular mortality (CM) reached as high as 6.8, 14.5, 43.5, and 15.3 percentage lung function decrement passive cigarette equivalents concerning the risk of school-aged children (PLFD), of LC, CM, PLFD, and LBW, respectively. and low birth weight (LBW)	
Phairuang et al. (2021) [48]	Bangkok, Central Thailand	Urban	November 2014–October 2015	PM_0.1-_bound 13 trace elements (Al, Cu, Cr, Fe, K, Ba, Ti, Ni, Mn, Na, Mg, Pb, Zn) linked to health risks in both non-carcinogenic and carcinogenic	The general population assesses the carcinogenic risk to be tolerable; however, during cold dry weather, the metal increase depends on biomass fires and meteorological conditions.
Phairuang et al. (2022) [100]	Southern Thailand	Suburban	January–December 2018	PM_0.1_-bound 13 trace elements (Al, Cu, Cr, Fe, K, Ba, Ti, Ni, Mn, Na, Mg, Pb, Zn) linked to health risks in both non-carcinogenic and carcinogenic	The non-carcinogenic and carcinogenic risks are measured to be in the tolerable risk assessment range in general populations in southern Thailand. However, during the pre-monsoon season, the metal levels increase depending on cross-border pollution and meteorological conditions.
Pothirat et al. (2021) [94]	Chiang Mai (North)	Urban	2016–2018	PMs associated with daily non-accidental mortality, including causes of death	Fine and coarse PMs were linked to daily non-accidental mortality on different lag days (1.009–1.018 and 1.016 for each 10 μg/m^3^ increment of coarse and fine PMs, respectively.
Punsompong et al. (2021) [16]	Whole Thailand	Urban	January–December 2016	Excess numbers of premature deaths linked to PM_2.5_ exposure linked to health endpoints i.e., stroke burden (SB), lung cancer (LC), ischemic heart disease (IHD), and chronic obstructive pulmonary disease (COPD)	Fine particles exposure related to premature mortality with displayed the most considerable burden in the central, northeast, north and south region (44%, 29%, 18%, 9%), respectively.
Sakunkoo et al. (2022) [81]	Khon Kaen (Northeast)	Rural	December 2020 and February 2021	Carcinogenic and non-carcinogenic health risks of PM_2.5_ related to heavy metals (Pb, Cu, Cd, Fe, Mn, Al, and Zn)	High carcinogenic risk in children 4.11 × 10^−2^ and the adult 7.70 × 10^−3^ in an agricultural zone
Singkam et al. (2022) [101]	Upper Northern	Urban	January 2014–December 2020	Effect of PM_2.5_ and meteorological parameters on the incidence rates of Chronic Obstructive Pulmonary Disease (COPD)	COPD patients increase the risk of COPD cases by 0.25% when PM_2.5_ by 1%.
Uttajug et al. (2021) [43]	Upper Nortnern	Urban	2014–2018	PM_10_ levels and hospital visits by children to expose respiratory disease, dermatitis, and conjunctivitis.	Increased 10 μg/m^3^ in PM_10_ on a burning day was associated with respiratory disease-related hospital visits.
Uttajug et al. (2022) [44]	Upper Nortnern	Urban	January to April 2014–2016 (before ban enforcement) and January to April 2017–2018 (after ban enforcement)	PM_10_, numbers of active fires, and age-standardized rates of hospital visits for respiratory diseases before and after ban enforcement	PM_10_ level and active hotspots reduced after ban implementation, ranging from 5.3 to 34.3% and 14.3–81.5%, respectively. In addition to hospital visits for respiratory diseases declined by 9.1%.
Vajanapoom et al. (2020) [45]	Chiang Mai (North)	Urban	From January 2002 and December 2016	PM_10_, gaseous pollutants (NO_2_, SO_2_, CO, and O_3_), and climatology in Chiang Mai and daily mortality counts	Reduced episodic PM_10_ levels have led to a decline in short-term all-cause mortality.
Wunnapuk et al. (2019) [102]	Chiang Mai (North)	Rural	March 2016 (PM_10_ > 50 μg/m^3^) and August 2016 (PM_10_ < 50 μg/m^3^)	DNA-damaged cells, cells with cytokinetic defect, and different types of cell death using a buccal micronucleus cytometry assay	Frequencies of buccal cells with micronuclei and binucleated cells were higher during months of high pollution (March), particularly in COPD patients, indicating DNA damage and instability

Kongpran et al. (2021) [96] estimated the carcinogenic risk from PAHs for subjects in the northern part of Thailand (Table 1). The harmful effects of PAHs are dependent on the mechanism of exposure. Benzo[a]pyrene (BaP) is a well-known PAH that causes cancer on a laboratory scale with long term exposure [24,31]. BaP-TEQ is a broadly used indicator to evaluate the extent of exposure to PAHs on human health, especially from biomass combustion in Thailand. Mueller et al. (2020, 2021) [97,98] reported a health risk assessment of the population in northern Thailand for a long period of time. They found that an increase in the level of coarse particles during biomass episodes in the north of Thailand was related to symptoms including ischaemic heart disease (IHD), chronic lower respiratory disease (CLRD), cerebrovascular disease (CBVD) and low birth weight (LBW) in infants.

Moreover, studies of metal and trace elements in PMs by Niampradit et al. (2022) [99] in the northern part (fine particles), Sukunkoo et al. (2022) [81] in the northeast part (PM_2.5_), and Phairuang et al. (2021, 2022) [48,100] in BMR and southern part (ultrafine particles) have been reported. These results indicate that toxic elements that are produced during biomass burning are still at a safe level regarding risk to human health. However, some elements have increased sharply during periods of high PM levels from BB, which should be a human health concern.

Punsompong et al. (2021) [16] reported on biomass fire episodes that affected human health in Thailand. They found that exposure to PM_2.5_ placed a considerable burden in the central region (44%), followed by the northeast (29%), north (18%), and south (9%), respectively. In the same manner, Pani et al. (2020) [22] reported on estimates of the health risk of equivalent black carbon in the northern part of Thailand during strong haze episodes. The findings indicated that there were serious health effects, some as high as passive cigarette equivalents.

The increasing level of PM_10_ and PM_2.5_ concentrations in the upper part of Thailand were found to be related to hospital visits by both children and adults. Several studies during haze and non-haze periods suggested that the general population in the northern part of Thailand, these periods are with respiratory disease-related hospital visits [[43], [44], [45],^101^]. Based on studies by Wunnapuk et al. (2019) [102], it appears that PM_10_ can affect COPD patients, indicating DNA damage and instability during high periods of pollution (March). However, respirable particles (PM_2.5_ and ultrafine particles (PM_0.1_)) pose a higher risk for human health problems [103]. The concern should be more focused on smaller particles from biomass burning that influence the transportation of polluted PM, which is vital on a multi-provincial scale (100–200 km) in Thailand [2].

## 5 Perspectives and new challenges for studies of biomass burning in Thailand

### 5.1 Size-fractionated PMs

Atmospheric particulate matter (PM) from biomass fires can have various chemical compositions with many sizes and characteristics [31,104]. Long-term exposure to high levels of ambient particles can cause a variety of human health problems, including irregular heartbeat, nonfatal heart attacks, decreased lung function, increased respiratory symptoms, and lung cancer [42,97,98]. Numerous epidemiological studies have confirmed the adverse health effects of atmospheric PM deposited in the human respiratory tract [94]. The deposition of a particular particle in the human respiratory tract depends on its aerodynamic diameter. Particle sizes with diameters between 10.0 and 4.7 μm are mainly deposited in the upper respiratory tract (nasal passage and pharynx) and can be eliminated by sneezing and coughing. Particle sizes between 4.7 and 2.1 μm are primarily deposited in the primary bronchi, while particle sizes smaller than 2.1 μm are deposited in respiratory bronchi and alveoli. Particle sizes lower than 0.43 μm can be deposited in alveoli where gas exchange occurs. These particles can affect gas exchange in the lungs and actually penetrate through the membrane into the circulatory system [105]. Interestingly, PM_0.1_ can enter the blood circulation and has a high potential for being ingested by cells [3]. Information on ambient nanoparticles is insufficient and health risks due to their small size and the challenge associated with collecting them under ambient conditions for studies of their actual physicochemical characteristics [106].

The cascade impactor sampler is the most widely used sampler for collecting size-fractionated PMs in ambient air [107]. However, artifacts due to the evaporation of semi-volatiles from conventional types of size-specific particle samplers, e.g., low-pressure impactors and Micro-orifice Uniform Deposit Impactor (MOUDI), continue to affect studies of the ambient particles that are produced during biomass episodes [28]. The new technology for size-classified PMs is also crucial for investigating the chemical and physical characteristics of PMs under ambient conditions in the atmosphere of Thailand. The retention or removal of semi-volatile particles for smaller particles down to the nano-size range can be volatilized within the sampling arrangement due to their large specific surface areas [108]. Taking into account that small particles related to chemical components with minimal artifacts during air sampling caused by the degradable characteristics of chemicals and semi-volatile substances are also a crucially vital issue in collecting size-fractionated particles [109].

### 5.2 Emission inventory analysis from biomass burning

The emission inventory (EI) estimates particulate pollution over a specific time period and location with uncertain emissions from some parameter in the calculation [12]. The uncertainty of each pollutant emission can be attributed to 2 leading causes: (1) the area that is burned and the dry biomass burn fraction that is burned as well as (2) the emission factor and default value in the EI calculation [79].

#### 5.2.1 Burned area

Two approaches have recently been reported to estimate burned areas using national crop production data and satellite data [79]. The national agricultural-specific data are available from many countries, and this information is used for particular distributions [110,111]. In Thailand, however, the satellite method frequently results in underestimates because the resolution of satellites that pass over Thailand is small or medium and is intended to capture information concerning the occurrence of fires [73].

Satellite image data is a valuable tool for monitoring biomass-burning events in Thailand [12,20]. The use of satellite imagery can provide information on the time, location and the extent of fires as well as the plant type that is being burned [18]. This information can help manage and monitor open fires and track land use and changes in land use over time [16]. In addition, satellites can be effectively used to measure the PM mass concentration generated by fires, which is vital for our understanding of the influence of open fires on the atmosphere and climate change [21,60,62]. Nevertheless, it is essential to note that it has to be combined with other data sources, including ground measurement and mathematical modeling, to obtain a complete picture of the situation [12].

For example, MODIS, one of the more famous satellite information systems for detecting and analyzing open biomass burning, is taking resolutions of 500 m or 1000 m in many countries. Even though the burning of residues in Thailand is mainly agricultural, rice fields usually have a small area of about 100 m, and only a short time (a few hours) is needed for the burning to reach completion [12]. In this case, there is a high probability that crop residue burning in Thailand is not detected by an orbiting satellite orbit. For this result, the authors recommend using country-specific data when available and decreasing the burned area uncertainty by using satellite data with a higher resolution in the future.

#### 5.2.2 Emission factors and other default parameters

Emission Factors (EFs) and other parameters that are used for calculating dry biomass burning are different for each crop type and geographical area of plant cultivation. EIs, incur a high uncertainty in evaluating air pollutants in each area. On a global scale, the present emission guide is used for estimating EIs at the national level. These include the Intergovernmental Panel on Climate Change (IPCC) report 2021 for greenhouse gas emission inventory. The Global Atmospheric Pollution Forum Air Pollutant Emission Inventory (GAFP) forum for all toxic air and greenhouse gases data manual 2012, and so on [112]. This emission factor and the default values are general for many EIs that are related to open biomass burning. However, for accurate data, country-specific values are needed to apply the data to Thailand. The primary EFs from Thailand to estimate EIs prefer land use. When EFs or default parameters are unavailable for Thailand, other Asia countries are the first choice, followed by others that have similar climates and agricultural crop cultivation [49]. There are available PM_2.5_ emission factors and chemical compositions from biomass combustion in Thailand [113,114] and PMs down to PM_0.1_ from Samae et al. (2021, 2022) [25,26].

### 5.3 Low-cost sensors and interpolation model for PMs monitoring

The PMs concentration has been regularly monitored by the Pollution Control Department (PCD) of Thailand. The PM_10_ and PM_2.5_ measurement methods of the PCD monitoring stations are based on US-EPA standard method (Federal Reference Method and Federal Equivalent Method), which consumes high investment and maintenance costs. Therefore, the number of stations and spatial coverage is still limited. In contrast, low-cost monitoring sensors are widely deployed and can be rapidly expanded all over the country because they are portable and easy to use and operate. Most low-cost sensors (LCS) detect PMs using an aerosol spectrometer based on a light-scattering method. Although the credibility of LCS data is a concern in Thailand, LSC's PMs monitoring network provides valid local data for air pollution management plans and can complement the standard stations [115,116]. Many studies have evaluated LCS compared with the traditional method and found that LCS can be an operational and reliable method for PMs measurement in addition to the traditional method [13,117]. PMs sensors are widely applied for IoT systems and modified with other sensors to measure air pollutants and weather [118]. Kanabkaew et al. (2019) [119] set a network of PM_2.5_, wind speed, and wind direction sensors for a haze early warning system in Thailand. Chunitiphisan et al. (2018) [117] applied LCS with Unmanned Aerial Vehicle (UAV) to monitor the moisture in 3D displays.

The estimation model is broadly implemented to monitor PM concentration and overcome the low coverage of air quality stations. The remote-sensing method and machine learning techniques are cost-effective ways to understand and predict PMs behavior, especially biomass-burning episodes in Thailand during the dry season [120,121]. Satellite data combined with ground data like PCD stations or low-cost sensors can generate an accurate PM_2.5_ concentration map for continuous and real-time monitoring [[122], [123], [124]].

## 6 Policy recommendation and air quality management

### 6.1 Prescribed burning in forest fuel management

The riskiest forest fires occur in Thailand's dry deciduous forests [125]. Leaf litter and other arid parts of trees accumulate in the ground in the woods during the dry season (December–April). Forest fires, therefore also occur in the dry season, depending on the available fuel. According to the Forest Fire Control Division (FFCD) (2019) [126], the main reasons for forest fires during 2016–2018 are the collection of forest products by local workers, i.e., mushrooms and bamboo (62%), hunting (10%), slash and burn (4%), animal farming (1%), conflicts (0.6%), incidents (0.3%), illegal logging (0.2%) and others (20%). These data suggest that most recorded forest fires are clearly related to human activities, particularly agricultural activities. These possibilities result in serious concerns regarding the atmospheric environment and public human health risks because many PMs are released from open burning in fields in croplands and in related forest fires.

The forested area, especially in northern and western Thailand, produces a substantial amount of leaf litter during the dry season. It may be necessary to dispose of some parts by incineration, which must be carried out at the right time and place [127]. This will help reduce damage from forest fires, preserve forest conditions, and reduce smog caused by uncontrollable forest fires. In many countries, prescribed burning is the most effective method for forest fire management. Banning forest fires without appropriate management options is unlikely to alleviate the burning problem in forest areas [128,129]. Although efforts are currently being made to recycle, reuse and reduce forest residues. Fire barriers are always used in large forest areas, but it is difficult to control and manage these forest areas. Interestingly, Yabueng et al. (2020) [130] reported that implementing a zero-burning policy in northern Thailand can decrease open-burning activities. However, the levels of fine particles from biomass fires have been reduced, but prolonged periods of smoke haze still exist. The policy for restraining open fires was extended from 2 months (mid Feb-mid April) to approximately a 3-month-long period (mid Feb-mid May), and the PM_2.5_ fraction was decreased during that period. Therefore, a prescribed burning policy can reduce open burning events during the policy implementation.

### 6.2 Biomass valorization

The aforementioned description indicates that biomass or lignocellulosic biomass waste typically generates particulate matter, which causes several air pollution problems. However, this material could be valorized into high value-added products, including biofuels, biochemicals, and biomaterials [131]. This valorization process could also alleviate the CO_2_ and PM emissions due to the direct burning of an enormous amount of biomass, thus provoking air pollution.

The biomolecular constituents of biomass are present in the form of lignocellulosic material, including cellulose, hemicellulose, and lignin, which could potentially be converted into valuable products via an integrated biorefinery system [132]. A biorefinery is typically a collectional process of separation, isolation, conversion, and purification. Several well-known biorefinery energy products, such as bioethanol, can be blended with conventional gasoline, which significantly reduces CO_2_ via combustion by 20% [133]. Biohydrogen and biogas produced from lignocellulosic biomass are other bioenergy resources that can serve transportation applications. Biochemicals produced via the biorefinery process can also be a product that is competitive with petroleum refineries due to their bioactive molecules in lignocellulosic biomass. Moreover, a biorefinery process can provide a wide range of intermediate building blocks and marketable products, similar to petroleum refinery products, including bioplastics, cosmeceuticals, biomaterials, and bio-based products [134]. Lignocellulosic biomass valorization is generally comprised of several processes, including pretreatment, hydrolysis, and bioconversion, which allows the transformation of the lignocellulosic biomass (agricultural wastes, forestry wastes, and industrial wastes) into more valuable products.

The development of integrated biorefinery processes for utilizing lignocellulosic biomass is economically viable and technologically feasible for industrial applications [135]. The valorization of lignocellulic biomass to green and clean energy is an alternative and potential solution, which can not only advance the utilization of bio-wastes but also meet the current carbon-neutral concept, indicating a great application potential in the new era of energy. Furthermore, biomass utilization could integrate with the circular-bioeconomy that maximizes the use of bioresources waste with an adequate mass flow within the entire value chain. In addition, the demand for such materials is increasing globally each day with the transition to a low-carbon economy and decreasing biomass burning in agricultural countries such as Thailand.

## 7 Conclusions

In the past decade, air quality in Thailand has been affected by biomass combustion. In the northern and northeastern regions of Thailand, open biomass burning has had an essential role in air quality in the past decade. However, in the Bangkok Metropolitan Region (BMR) the main contributor to air pollutants are motor vehicles and biomass burning. In Southern Thailand, the impact of maritime aerosols, biomass combustion, and possible agricultural residue burning are the primary sources. However, studies on the spatiotemporal characteristics of atmospheric particles in Thailand are lacking, especially studies related to the ultrafine size range. Consequently, the physical and chemical characteristics of ultrafine particles in typical cities of Thailand need to be further investigated for the primary emission sources and air quality. Policy recommendations, such as prescribed burning in conjunction with forest management, and biomass utilization, play an essential role in air quality management in Thailand. Data based on our current knowledge of the spatial and temporal variation of biomass-derived PM in Thailand will benefit air quality management, which leads to a critical global warming problem and adverse impacts on public health in developing countries.

## Author contribution statement

All authors listed have significantly contributed to the development and the writing of this article.

## Funding statement

This work was financially supported by the Office of the Permanent Secretary, Ministry Higher Education, Science, Research and Innovation in Thailand (Grant No. RGNS 63-253). Moreover, this work was partially supported by JICA-JST SATREPS (Grant No. JPMJSA2102), JSPS KAKENHI 21H03618, and Sumitomo Foundation, Japan.

## Data availability statement

The data that has been used is confidential.

## Declaration of interest’s statement

The authors declare no competing interests.
